# Diphlorethohydroxycarmalol Derived from *Ishige okamurae* Improves Behavioral and Physiological Responses of Muscle Atrophy Induced by Dexamethasone in an In-Vivo Model

**DOI:** 10.3390/pharmaceutics14040719

**Published:** 2022-03-27

**Authors:** Bomi Ryu, Seyeon Oh, Hye-Won Yang, Batsukh Sosorburam, Dong-Min Chung, Minyoung Seo, Shin-Jae Park, Kyunghee Byun, You-Jin Jeon

**Affiliations:** 1 Department of Marine Life Science, School of Marine Biomedical Sciences, Jeju National University, Jeju 63243, Korea; swfft@jejunu.ac.kr; 2 Functional Cellular Networks Laboratory, Department of Medicine, Graduate School, Lee Gil Ya Cancer and Diabetes Institute, Gachon University, Incheon 21999, Korea; seyeon8965@gmail.com (S.O.); sosorburam72@gmail.com (B.S.); 3 Department of Anatomy & Cell Biology, College of Medicine, Gachon University, Incheon 21936, Korea; 4 Shinwoo Co., Ltd., Jinju 52839, Korea; jdm@shinwoocorp.com (D.-M.C.); min086@shinwoocorp.com (M.S.); sjpark@shinwoocorp.com (S.-J.P.); 5 Marine Science Institute, Jeju National University, Jeju 63333, Korea

**Keywords:** muscle atrophy, *Ishige okamurae* (IO), diphlorethohydroxycarmalol (DPHC), dexamethasone (DEX), physical function, metabolic property

## Abstract

Muscle atrophy refers to the loss of skeletal muscle mass, myofiber size, and related physical functions such as walking speed or grip strength caused by aging or a lack of physical activity due to injury or illness and can also be attributed to excessive exposure to corticosteroids. *Ishige okamurae* (IO) and its active component, diphlorethohydroxycarmalol (DPHC), have been known to improve glucose homeostasis by controlling the contraction of skeletal muscles. Based on this idea, we hypothesized that the effects of DPHC and IO extract on muscle metabolism are associated with their role in improving muscle physical function. This study assessed the effects of DPHC or IO extract on muscle behavioral responses with their metabolic properties in muscle atrophy induced by glucocorticoids and dexamethasone (DEX) in vivo. In addition to the improvement in muscle behavioral response by DPHC or IO extract, the loss of muscle fiber and the related metabolic properties by DEX exposure in the gastrocnemius and soleus of calf muscle was prevented. These findings suggest that IO extract and its active component DPHC can potentially prevent muscle atrophy caused by exposure to corticosteroids and could be used to treat reverse skeletal atrophy.

## 1. Introduction

Brown alga, *Ishige okamurae* (IO), and its prominent component diphlorethohydroxycarmalol (DPHC) have been studied in various rodent models to investigate their therapeutic activity against type 2 diabetes mellitus, hyperlipidemia, and hypercholesterolemia, due to their exceptional oral bioavailability [1,2]. Additionally, their activity on glucose homeostasis in skeletal myotubes has been reported earlier, suggesting that IO extract and its active component DPHC could activate glucose transport into the skeletal muscle, thereby improving glucose homeostasis and inducing energy expenditure by muscle contraction [3]. In addition to these reports of DPHC acting on the skeletal muscle, a recent investigation has reported its benefits against the inflammatory response of myopathy in skeletal muscle [4,5,6]. These findings suggest a potential role of IO extract and its active component DPHC in facilitating muscle response in muscle atrophy.

Muscle atrophy refers to the loss of skeletal muscle mass, myofiber size, and related functions such as walking speed or grip strength due to aging or a lack of physical activity caused by an injury or illness; it can also be attributed to excessive exposure to corticosteroids [7,8]. Muscle mass in humans decreases at a rate of approximately 3–8% per decade after the age of 30; the rate of decline is even higher after 60 years of age [9,10]. The loss of muscle mass reaches 30% in the 70 s compared to that in 30 s or 40 s. Changes in muscle mass are also accompanied by a progressive increase in fat mass and consequent changes in body composition [11]. This in turn has various implications on several conditions, including diabetes, obesity, heart disease, and osteoporosis [11]. Muscle atrophy has also been shown to affect the prognosis of cancer or surgery, leading to the recognition of sarcopenia as a disease with the ICD-10-CM code of M62.84 in October 2016 (www.prweb.com-prweb13376057, Washington, D.C. 28 April 2016) [12]. This designation has accelerated the interest of physicians diagnosing sarcopenia as well as pharmaceutical companies in developing pharmaceuticals for treating sarcopenia.

Glucocorticoids are endocrine hormones secreted from the adrenal cortex that are reportedly crucial in protein degradation and synthesis in the ubiquitin proteasome pathway of skeletal muscle [13]. However, excess levels of glucocorticoids can activate the glucocorticoid receptor, which mediates the cellular response associated with skeletal muscle atrophy. It reduces protein synthesis and accelerates protein degradation resulting in the negative balance of catabolic muscle protein metabolism [14]. Following exposure to increased glucocorticoid levels, muscle atrophy by means of protein degradation is induced mainly through the activation of the transcription factor forkhead box O3 (Foxo3) as well as the upregulation of the expression of muscle-specific ubiquitin ligases [15]. Particularly, in muscle atrophy, transcript levels of Foxo3 and its main regulators, muscle-specific ubiquitin ligases, such as atrogin-1 and muscle ring finger 1 (MuRF1), are highly upregulated due to the presence of glucocorticoids [16,17].

The synthetic glucocorticoid dexamethasone (DEX) has been used as an anti-inflammatory and immunosuppressive agent with a relatively long-term biological activity of 36–54 h. However, high doses and prolonged use have been reportedly associated with reduced protein synthesis and loss of muscle mass [18,19]. Consequently, DEX has been extensively studied to understand the mechanism underlying muscle atrophy in rats, mice, and other in vitro models [20,21].

We investigated whether using IO extract or its active constituent DPHC in a DEX-induced skeletal muscle atrophy-in vivo model normalizes changes in skeletal muscle function, including endurance and strength. We also explored the effects of IO extract and DPHC on various muscle fiber types and examined their metabolic properties in an in vivo muscle atrophy model. In addition, we explored the underlying signaling mechanisms. Data from this study provide evidence supporting the beneficial effects of DPHC containing IO extract, which is known to promote energy expenditure in skeletal muscle, on muscle function and its related cellular responses in muscle atrophy caused by glucocorticoid and DEX exposure. The study further provides insights to design strategies for developing pharmaceuticals to improve the prognosis of patients with muscle atrophy.

## 2. Materials and Methods

### 2.1. Materials

Marine algae species *Ishige okamurae* (IO) was collected from the shores of Jeju Island in April 2020. The samples were immediately washed with running water to remove epiphytes, dried, and ground into a powder. Thereafter, the powder was extracted in 50% ethanol (*v*/*v*, in water) under stirring for 24 h at room temperature, then it was filtered. The filtrated extract was concentrated under decompression (−500~600 mmHg) and spray-dried (Shinwoo Co., Ltd., Lot No. SW8D10SA, Dongan-gu, Anyang-si, Gyeonggi-do, Korea). Briefly, IO used in this study was standardized on the assumption of diphlorethohydroxycarmalol (DPHC, CAS 138529-04-1) by an HPLC analysis method. DPHC in IO was verified by using quadrupole time-of-flight liquid chromatography-mass spectrometry (Q-TOF LC-MS/MS) using an electrospray ionization (ESI) source (maXis-HD, Bruker Daltonics, Breman, Germany) at the Korea Basic Science Institute (KBSI; Ochang, Korea), equipped with an Agilent poroshell 120 EC-C18 column (4.6 mm × 100 mm, 4 μm). The mobile phase consisted of (A) 0.1% formic acid in water and (B) ACN containing 0.1% formic acid. The HPLC eluting conditions were as follows: 20% to 40% B for 30 min followed by 10 min re-equilibration time of the column. Spectra were acquired in continuum and positive mode for [M + H]^+^ at the *m*/*z* 513. For the quantification of DPHC in IO, DPHC was purchased from Aktin Chemical Inc. (98% purity, Chengdu, China). The DPHC content in the IO extract was 2.41% (Figure S1). DEX was obtained from Sigma-Aldrich (D2915, Sigma-Aldrich, St. Louis, MO, USA). Decanoate, used as a positive control in this study, was purchased from Organon Pharmaceutical Company (Deca-Durabolin, Organon, São Paulo, Brazil).

### 2.2. Dex-Induced Muscle Atrophy Model

Male CrljOri:CD1 (ICR) mice at 9 weeks of age were obtained from OrientBio Inc. (Seongnam, Korea). The animals were maintained under controlled conditions (room temperature, 23 ± 2 °C; relative humidity, 50 ± 5%; 12 h light/dark cycles) with ad libitum access to water and a regular rodent chow diet. Animals were randomized into six groups with 10 mice per group after 1 week of acclimatization: (1) the vehicle-control group that received saline (control), (2) the DEX-injected group (DEX-saline), (3) DEX-injected and 2.41 mg/kg/day of DPHC administered group, (4) DEX-injected and 50 mg/kg/day of IO extract administered group, (5) DEX-injected and 100 mg/kg/day of IO extract administered group, (6) DEX-injected and 200 mg/kg/day of IO extract administered group, and (7) DEX-injected and 5 mg/kg/day of decanoate. The experimental protocols were approved by the Institutional Animal Care and Use Committee of Gachon University (Approval No. LCDI-2020-0003, 2020-04-13).

DPHC or IO extract was dissolved in distilled water, and the control group was administered the same volume of saline. DPHC or IO extract was administered by oral gavage every day for 28 days prior to DEX injection and maintained throughout the experimental period of 38 days. DEX (1 mg/kg body mass) was injected subcutaneously once daily for 10 days to induce muscle atrophy.

Muscle mass was quantified using an automatic electronic balance and normalized by the relative total weight (% of body weight) to minimize differences among individual animals in terms of body surface area. Bodyweight, fat, and lean body mass in grams of all mice were measured using a nuclear magnetic resonance analyzer (Mini-spec, LF-90II, Bruker Optics GmbH, Ettlingen, Germany). Bodyweight and food intake were assessed weekly.

### 2.3. Grip Strength

A grip strength meter (JD-A-22, JEUNGDO BIO& PLANT Co., Ltd., Seoul, Korea) was used to measure the force immediately before the mice fell off the grid by placing the mice on the grid and gently pulling their tails. Grip strength was measured on day 35 after administering DPHC or IO extract. The measured force was recorded 10 times at one-minute intervals, and the average force was considered for further analysis.

### 2.4. Ladder Climbing Exercise

The mice with weights attached to their tails were made to climb a 1 m ladder with a 2 cm grid inclined at 70°. The initial attached weight was 20% of their body weight, which was increased gradually to 100% of their body weight. Muscle strength was confirmed by measuring the time taken to reach the top of the ladder. When the mice reached the top of the ladder, they were allowed to rest in the dark for 1 min. The ladder climbing test was conducted in triplicates, and the average value was used for future calculations. The test mice were allowed to rest for 2 min between the sets.

### 2.5. Measurement of Calf, Gastrocnemius and Soleus Muscle Thickness

The thickness of the left hind calf, gastrocnemius and soleus were measured following muscle exposure after sacrifice using a ruler in mm, in order to decrease variability from the surrounding tissues.

### 2.6. Evaluation of Muscle Histopathology

The muscle tissues of mice stored in 4% paraformaldehyde (Sigma-Aldrich, Cat. No. 16005, St. Louis, MO, USA) were fixed at 4 °C for 12 h and embedded in paraffin using a tissue processor (Thermo Fisher Scientific, Waltham, MA, USA). The paraffin blocks were cut into 7-µm-thick sections using a microtome (Leica, Wetzlar, Germany), placed on a coated slide, and dried at 45 °C overnight. Hematoxylin & Eosin staining was used to determine the cross-sectional area (CSA) of the muscle fibers. Muscle tissue slides were incubated with hematoxylin (DAKO, Glostrup, Denmark) for 1 min, rinsed in distilled water for 10 min, and placed in eosin Y solution (Sigma-Aldrich, St. Louis, MO, USA) for 1 min at room temperature. Nuclei appeared in blue color, and the cytoplasm was colored light pink. The slides were observed under an optical microscope (Olympus Optical Co., Nagano, Japan). The mean muscle fiber CSA was calculated using ImageJ software 1.53j (NIH, Bethesda, MD, USA). Histological analyses were conducted in a blinded manner. Three operators conducted each analysis at least in triplicate.

### 2.7. Extraction of RNA and Quantitative Real-Time-Polymerase Chain Reaction (qRT-PCR)

The frozen muscle was homogenized with 500 µL of RNiso (Takara, Shiga, Japan) by pipetting, mixed with 0.1 mL chloroform (Amresco, Cleveland, OH, USA), and centrifuged at 12,000× *g* for 15 min at 4 °C. The acquired transparent layers were mixed with the same volume of isopropanol by centrifugation at 12,000× *g* for 15 min at 4 °C. The mixture was further centrifuged at 12,000× *g* for 15 min at 4 °C, and the isolated RNA samples were washed with 75% ethanol and dried. Subsequently, the RNA pellet was dissolved in diethyl pyrocarbonate-treated water and RNA was quantified while using a Nanodrop 2000 (Thermo Fisher Scientific, Waltham, MA, USA). cDNA was synthesized using a PrimeScript First Strand cDNA Synthesis Kit (Takara, Shiga, Japan). The primer was mixed with distilled water and then placed in 384-wells. Template (cDNA) and SYBR green (TAKARA) were subsequently added, and then validated using the CFX 384 Touch™ Real-Time PCR Detection System (Bio-Rad Laboratories, Irvine, CA, USA). The reaction efficiency and cycle threshold values were determined using CFX Manager™ software (Bio-Rad Laboratories, Irvine, CA, USA). The primer sequences for target genes are listed in Table S1.

### 2.8. Statistical Analysis

Non-parametric tests were performed in this study. The Kruskal–Wallis test was used to validate the significance of the differences between the seven groups. If a significant difference was confirmed by Kruskal–Wallis test, multiple comparisons were conducted using a Mann–Whitney U test as a post hoc test. Experiments were performed in triplicate per sample, and all results are presented as mean ± standard deviation. All statistical analyses were performed using IBM SPSS version 22 (IBM Corp., Armonk, NY, USA).

## 3. Results

### 3.1. IO Extract and Its Active Component DPHC Improved the Muscle Behavioral Response in DEX-Induced Skeletal Muscle Atrophy-In Vivo Model

**Figure 1:**
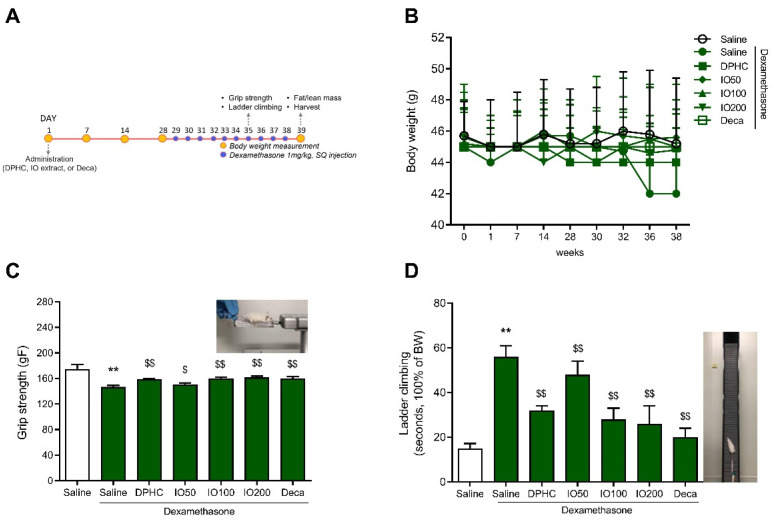
Schematic representation of the in-vivo study design (**A**). Nine-week-old ICR mice were administrated IO extract (50, 100, 200 mg/kg) or DPHC (2.35 mg/kg) daily for 38 days and DEX (1 mg/kg body mass) was injected subcutaneously daily for 10 days. Body weight was determined during the period of administration (**B**). Muscle behavioral responses were assessed using grip strength (**C**) or ladder climbing (**D**) measurements. The captured image for each performance is provided in the upper-right. Each group was examined in n = 7 mice. All values are expressed as the mean ± standard error. Significant differences are indicated as ** *p* < 0.01 compared with control or ^$^ *p* < 0.05, ^$$^ *p* < 0.01 compared with DEX-saline (Mann–Whitney U test).

We investigated whether the effect of IO extract and its active component DPHC on muscle behavioral response, including grip strength and ladder climbing, in DEX-induced skeletal muscle atrophy-in vivo model (Figure 1A). The mice were administered DPHC (2.41 mg/kg/day), decanoate (5 mg/kg/day), or IO extract (50, 100, 200 mg/kg/day) for 28 days and muscle atrophy by DEX injection (1 mg/kg/day) for another 10 days. Decanoate was used as a positive control in this study to increase muscle mass and strength [16]. As the result for bodyweight for the 10 days observed represented that no-adverse effect by DEX injection or DPHC or IO extract administration (Figure 1B).

At day 10, after muscle atrophy caused by DEX, the changes in the grip strength (gF) and the elapsed time (seconds) for ladder climbing in mice were investigated compared with those in the control group, which contained healthy mice. In the control group, the 175 g of grip strength significantly reduced to 147 g in the DEX-saline group (** *p* < 0.01, Figure 1C). However, in the DPHC or IO extract (100 or 200 mg/kg/day)-treated groups, grip strength had improved significantly (^$$^ *p* < 0.01) to 160 and 162 gF, respectively, which was similar to the positive group of 160 gF, highlighting the remarkable protective ability of IO extract and its active component DPHC against DEX-induced muscle atrophy. As shown in Figure 1D, the elapsed time (seconds) for ladder climbing in the DEX-saline group increased by approximately 56 s (** *p* < 0.01) compared to that in the control group of 15 s and detected a considerable shortening to 32 s following DPHC pretreatment. Additionally, pretreatment with IO extract at 50, 100, or 200 mg/kg/day significantly reduced the elapsed time to 48, 28, and 26 s, respectively.

### 3.2. IO Extract and Its Active Component DPHC Improved Calf Skeletal Muscle and Muscle Types in DEX-Induced Skeletal Muscle Atrophy in an In Vivo Model

**Figure 2:**
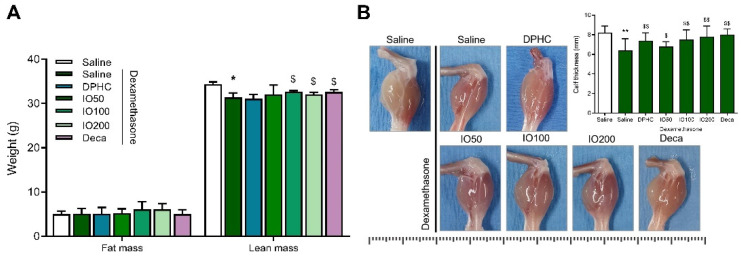
Fat mass and lean mass were determined on day 39 post-treatment (**A**). Representative calf muscle image and its thickness was assessed (**B**). All of the lines designating dimensions in ruler above each muscle indicate length in millimeters. The thickness was measured using a ruler and is expressed in millimeters. Each group was examined in n = 7 mice. All values are expressed as the mean ± standard error. Significant differences are indicated as * *p* < 0.05, ** *p* < 0.01 compared with control or ^$^ *p* < 0.05, ^$$^ *p* < 0.01 compared with DEX-saline (Mann–Whitney U test).

Following the muscle response of the mice in physical performance, we observed the changes in fat mass and lean mass, including muscle mass, as well as bones and bodily fluid of the mice induced muscle atrophy by DEX and in the group pretreated with DPHC, IO extract, or decanoate. Although no significant differences in fat mass were observed between the groups, the lean mass in the DEX-saline group showed a significant decrease (Figure 2A, * *p* < 0.05) compared to the control group. Meanwhile, the group administered IO extract (100 or 200 mg/kg/day) showed an improvement in lean mass, similar to the decanoate group. This result suggested that DEX used in this study caused a marked decline in lean mass, not body weight or fat mass, compared to the control group. In addition, IO extract preserved lean mass; however, no changes in lean mass were observed in the DPHC group.

In Figure 2B, consistent with the decrease in lean mass, DEX/Saline group showed a significant decrease to 6.4 mm in the thickness of calf muscle compared to that in the healthy mice (8.2 mm); interestingly, the group with DPHC administration showed remarkably increased tendency similar to the IO extract-group. These results suggest that DPHC or IO extract treatment preserved the calf muscles.

**Figure 3:**
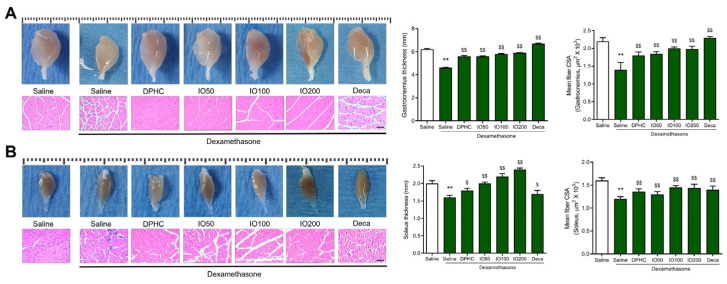
Representative gastrocnemius in calf muscle and its thickness were assessed in DEX-treated mice displaying muscle atrophy (**A**). H&E-stained section and cross-sectional area of gastrocnemius muscle fibers Representative soleus in calf muscle and its thickness were assessed (**B**). H&E-stained section and cross-sectional area of soleus muscle fibers. All of the lines designating dimensions in ruler above each muscle indicate length in millimeters. The thickness was measured using a ruler and is expressed in millimeters. Scale bars denote 100 μm. Each group was examined in n = 3 mice. All values are expressed as the mean ± standard error. Significant differences are indicated as ** *p* < 0.01 compared with control or ^$^ *p* < 0.05, ^$$^ *p* < 0.01 compared with DEX-saline (Mann–Whitney U test).

We further investigated the effects of DPHC or IO extract on the muscle type of calf muscle, gastrocnemius, or soleus muscle, which provide a significant propulsive force to different muscle movements, respectively. In the gastrocnemius muscle, the DEX-saline group had a 4.6 mm gastrocnemius thickness compared to that in the control group (6.2 mm). However, the DPHC and IO extract groups recovered to thicknesses of 5.6, 5.6, 5.8, and 5.9, respectively, which was similar to that in the decanoate group (^$$^ *p* < 0.01) (Figure 3A). Histologically, the gastrocnemius muscle fibers of the control group showed intimate contact to form muscle bundles that were wrapped by the perimysium (Figure 3A). In contrast, in the DEX/saline group, the perimysium was significantly decreased, resulting in a decrease in the mean fiber CSA in the gastrocnemius muscle, while the DPHC and IO extract groups had conserved histological alterations and their average fiber CSA of the muscle was also significantly elevated. In addition, the tendency of the changes treated by DPHC or IO extract in muscle thickness of the gastrocnemius muscle was observed to be similar to the average fiber CSA.

In the soleus muscle, which contributes to walking or static stands, the thickness in the DEX-saline group also exhibited excessive changes compared to that in the control group, while the groups administered with DPHC or IO extract showed the prevention of the loss of soleus muscle (Figure 3B). In particular, the group administered IO extract (100 or 200 mg/kg/day) showed a significant increase in the thickness of the soleus muscle compared to the control group. In addition, myofiber diameter significantly decreased in soleus muscles in the DEX-saline group compared to the control group, and this decrease was significantly improved in the DPHC or IO extract groups (Figure 3B). In addition to the changes in the gastrocnemius muscle, the soleus muscle was also protected by DPHC or IO extract, suggesting that DPHC or IO extract treatment can attenuate muscle atrophy induced by DEX.

### 3.3. IO Extract and Its Active Component DPHC Improved the Metabolic Properties of the Gastrocnemius Muscle in the DEX-Induced Skeletal Muscle Atrophy-In Vivo Model

**Figure 4:**
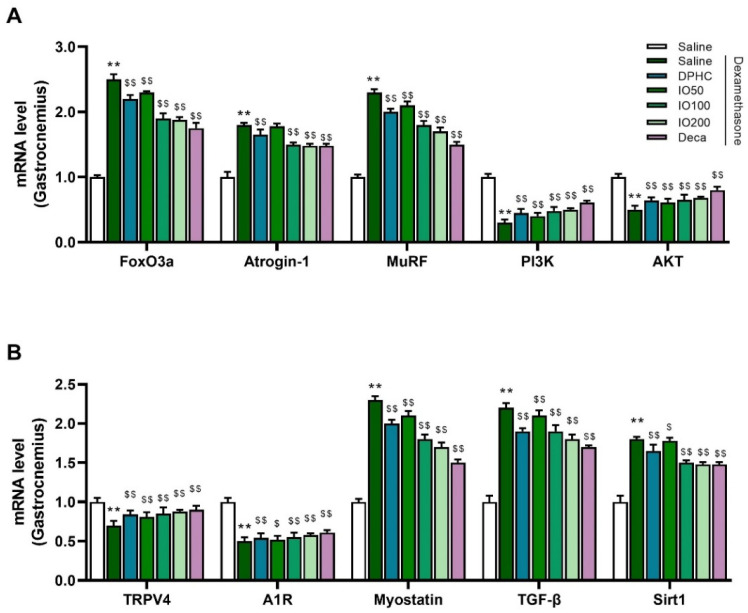
Levels of mRNA related to protein degradation and muscle growth in gastrocnemius muscle of DEX-treated mice displaying muscle atrophy. The panels display results for FoxO3a, Atrogin-1, MuRF, PI3K and AKT (**A**). TRPV4, A1R, Myostatin, TGF-β, and Sirt1 are aligned in (**B**). Each group was examined in n = 3 mice. All values are expressed as the mean ± standard error. Significant differences are indicated as ** *p* < 0.01 compared with control or ^$^ *p* < 0.05, ^$$^ *p* < 0.01 compared with DEX-saline (Mann–Whitney U test).

In muscle atrophy, the activation of the transcription factor FoxO3a modulates the expression of muscle-specific ubiquitin ligases, such as atrogin-1 and MURF1, which are involved in protein degradation in skeletal muscle [22,23]. To determine whether DPHC or IO extract regulates the physiological mechanisms associated with muscle atrophy in the gastrocnemius and soleus muscles, we examined the transcript levels of FoxO3a and muscle-specific ubiquitin ligases atrogin-1 and MuRF1, respectively. Treatment with DPHC or IO extract at 100 or 200 mg/kg/day markedly inhibited DEX-mediated increased expression of FoxO3a, atrogin-1, and MuRF1, in gastrocnemius muscle (Figure 4A). To further characterize the mechanisms mediating the effects of DPHC or IO extract on DEX-induced muscle atrophy models, we measured the phosphatidylinositol 3-kinase (PI3K)/Akt signaling pathway in the gastrocnemius muscle, a crucial pathway regulating protein synthesis and muscle hypertrophy. The results demonstrated that treatment with DPHC or IO extract restored the DEX-mediated reductions in PI3K and Akt mRNA levels in the gastrocnemius muscle (Figure 4A). These data indicated that DPHC or IO extract inhibited protein degradation by inactivating FoxO3a and muscle-specific E3 ubiquitin ligases (atrogin-1 and MuRF1) and increased protein synthesis through the activation of the PI3K/Akt pathway in the gastrocnemius muscle.

In addition, subcutaneous exposure to DEX significantly decreased the mRNA levels of TRPV4 and A1R in the gastrocnemius muscle, which may be associated with the contractile function of skeletal muscle [24,25]. As shown in Figure 4B, DPHC or IO extract upregulated TRPV4 and A1R mRNA levels compared with those in the DEX-saline group, providing direct evidence that DPHC or IO extract can facilitate muscle contraction and increase muscle growth by resisting DEX-induced muscle atrophy, which was comparable to those administered with decanoate. In addition, the elevation of the mRNA levels of myostatin, TGF-β, and Sirtuin 1 (Sirt1), a representative negative regulator of muscle growth, was significantly inhibited by treatment with DPHC or IO extract (Figure 4B). This result suggests that DPHC or IO extract upregulates the muscle-shielding effect via downregulation of SIRT1 and myostatin and can enhance muscle growth by activating A1R and TRPV4 in the gastrocnemius muscle. Overall, we observed that metabolic dysfunction in the gastrocnemius muscle upon DEX exposure led to enhanced ubiquitin-proteasome-mediated protein catabolism and reduced muscle growth/protein synthesis. DPHC or IO extract improved muscle/protein metabolism of gastrocnemius muscle and attenuated DEX-induced muscle atrophy by reversing the dysfunction.

Furthermore, the responses to oxidative stress in the gastrocnemius muscle in the DEX-saline group, followed by DPHC or IO extract treatment, were measured. The group exposed to DEX in the gastrocnemius muscle was shown to stimulate inducible nitric oxide synthase (iNOS), interleukin-1β (IL-1β), and tumor necrosis factor-α (TNF-α), and reduced the mRNA levels of the antioxidative gene, glutathione Peroxidase 4 (GPx4). In contrast, the groups administered DPHC or IO extract showed reverse changes, comparable to decanoate (Figure S2).

### 3.4. IO Extract and Its Active Component DPHC Improved the Metabolic Properties of Soleus Muscle in DEX-Induced Skeletal Muscle Atrophy-In Vivo Model

**Figure 5:**
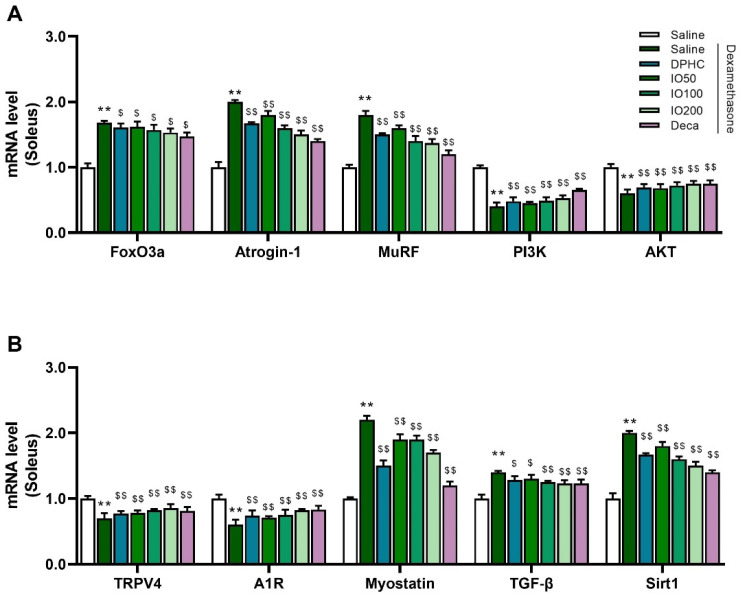
Levels of mRNA related to protein degradation and muscle growth in soleus muscle of DEX-treated mice displaying muscle atrophy. The panels display results for FoxO3a, Atrogin-1, MuRF, PI3K, and AKT (**A**), TRPV4, A1R, Myostatin, TGF-β, and Sirt1(**B**) are aligned in (**B**). Each group was examined in n = 3 mice. All values are expressed as the mean ± standard error. Significant differences are indicated as ** *p* < 0.01 compared with control or ^$^ *p* < 0.05, ^$$^ *p* < 0.01 compared with DEX-saline (Mann–Whitney U test).

Changes in the transcript levels of muscle atrophy-related genes in the soleus muscle were also observed (Figure 5). The group exposed to DEX in the soleus muscle stimulated the E3 ubiquitin ligases atrogin-1 and MuRF-1 by increasing FoxO3a and downregulating the PI3K and Akt signaling pathway atrophy-related genes, while DPHC or IO extract treatment reversed these changes. DPHC or IO extract inhibited intracellular protein degradation of the soleus muscle following DEX exposure, as indicated by the marked decrease in atrogin-1 and MuRF-1 levels with FoxO3a (Figure 5A). Moreover, DPHC or IO extract enhanced PI3K and Akt levels, thereby inducing protein synthesis (Figure 5A).

The additional influence of DPHC or IO extract treatment on the transcript levels of TRPV4 and A1R and its negative regulator, myostatin, TGF-β, and Sirt1, which contribute to muscle growth/degradation in soleus muscle, was explored (Figure 5B). The suppressed levels of TRPV4 and A1R in the DEX-saline group were significantly improved following treatment with DPHC or IO extract, which was similar to that in the decanoate group (^$$^ *p* < 0.01). As expected, dysfunction of muscular protein degradation-related genes, myostatin, TGF-β, and Sirt1 was remarkably recovered after treatment with DPHC or IO extract compared to the DEX-saline group, suggesting that DPHC or IO extract could mitigate the upregulated muscle protein degradation-related genes and strengthen the downregulated muscle protein growth-related genes resulting from DEX exposure in the soleus muscle.

In addition, similar to the changes in the gastrocnemius muscle, a significant increase in oxidative stress-related genes (iNOS, IL-1β, and TNF-α) in the soleus muscle following DEX exposure decreased in the DPHC or IO extract treatment group. In addition, the reduction of the antioxidative gene GPX-4 in the DEX-saline group significantly improved following treatment with DPHC or IO extract (Figure S3).

## 4. Discussion

Skeletal muscle, which accounts for up to 50% of the total body mass, plays a crucial role in physical activity and energy metabolism and is responsible for maintaining whole-body metabolic homeostasis [26]. Excessive loss of muscle mass can compromise physical function, impair the efficacy of many different treatments, and increase morbidity and mortality [8]. Therefore, many studies are being conducted to develop effective and safe therapeutic avenues for muscle atrophy [27,28,29,30]. Various in vitro and in vivo studies on the effects of natural products and medicinal plants on muscle atrophy under various conditions have received increased attention [31,32,33].

Our hypothesis is based on previous reports which suggested that IO extract and its active component DPHC can enhance glucose homeostasis and that the protein level is related to muscle contraction and improves myopathy in skeletal muscle cells in vitro [3,5,6]. Although the potential of IO extract and its active component DPHC for treating muscle atrophy has been suggested, an in vivo study of skeletal muscle atrophy to evaluate the effect of IO extract and its active component DPHC and its related properties in skeletal muscle function is needed. Accordingly, this study was conducted to verify the effects of DPHC or IO extract treatment on muscle atrophy induced by glucocorticoids and DEX in vivo.

The mice exposed to DEX showed lower muscle behavioral response through decreased grip strength and endurance by extended time to climb the ladder. Paralleling the changes in muscle behavioral response, the lean mass and thickness of calf muscle in the DEX-saline group decreased compared to that in the control group. Interestingly, oral administration of DPHC or IO extract improved the physical activity of muscle atrophy induced by DEX exposure.

The beneficial effects of DPHC or IO extract were also observed in calf muscle thickness, gastrocnemius muscle, and soleus skeletal muscle. Skeletal muscle fibers can be divided into gastrocnemius (a representative fast-twitched white muscle) and soleus (a representative slow-twitched red muscle) muscles; generally, it is known that gastrocnemius muscle fiber has relatively related to contractile force with a high capability for electrochemical transmission of action potentials whereas soleus muscle fiber can support prolonged aerobic physical activity and increased fatigue resistance [30,34]. Consistent with the results in the calf muscle, we observed that administration of DPHC or IO extract prevented the loss of myofiber CSA in the gastrocnemius and soleus muscles following DEX exposure. This evidence suggests that DPHC or IO extract could protect the gastrocnemius and soleus in calf muscle against DEX exposure and closely correlate with enhanced muscle behavioral response, such as grip strength and endurance.

The weakened muscle mass and strength of the group exposed to DEX are related to an imbalance in protein synthesis and degradation by activating the proteolytic system [35,36]. In turn, the overwhelmed proteolytic system further accelerates the degradation of skeletal muscle proteins [31]. The muscle-specific ubiquitin ligases atrogin-1 and MuRF-1, which increase FoxO3a transcript levels, are implicated in protein degradation in skeletal muscle [22,23]. The ubiquitin–proteasome-dependent proteolytic pathway can be suppressed by promoting the PI3K and Akt signaling pathways [37,38]. In addition, the adenosine receptor A1R and a member of the TRP channel superfamily TRPV4 are involved in the physiological functions of skeletal muscle and are closely associated with muscle growth [20]. Myostatin, TGF-β, and Sirt1 have been reported to act on muscles to stimulate muscle degradation in muscle atrophy caused by DEX exposure [39,40]. Interestingly, we observed that the effects of DPHC or IO extract in muscle atrophy caused by exposure to DEX in both gastrocnemius and soleus muscles were involved in increased transcript levels for protein synthesis and muscle growth activation but decreased the transcript level for protein degradation and muscle growth inhibition.

The metabolic properties and their mechanism related to protein synthesis and muscle growth following the administration of DPHC or IO extract showed a correlation, helping us understand the result as a change in the muscle behavioral response. Therefore, DPHC or IO extract may help prevent loss of muscle from DEX-induced muscle atrophy by coordinating protein synthesis and muscle growth.

## 5. Conclusions

This study shows that IO extract or its active component DPHC can improve the muscle behavioral response by preventing the loss of muscle fiber and preventing the changes in the related metabolic properties of the gastrocnemius and soleus muscles after DEX exposure in an in vivo model of muscle atrophy. The mRNA transcript levels indicate that IO extract or its active component DPHC effectively intervene in the process of muscle fiber loss by effectively ameliorating the dysfunction of protein degradation/synthesis and muscle growth in both gastrocnemius and soleus muscles. Therefore, IO extract and its active component DPHC may be a potential therapeutic agent for treating DEX-induced muscle atrophy.

## Data Availability

Not applicable.

## Supplementary Materials

The following supporting information can be downloaded at: https://www.mdpi.com/article/10.3390/pharmaceutics14040719/s1, Figure S1. HPLC (A) and MS/MS (B) chromogram data of DPHC in IO and standard curve data for quantification of DPHC (C). Figure S2: Levels of mRNA related to oxidative stress in gastrocnemius muscle of DEX-treated mice displaying muscle atrophy; Figure S3: Levels of mRNA related to oxidative stress in soleus muscle of DEX-treated mice displaying muscle atrophy; Table S1: List of primer for qRT-PCR.
