# Supplementary material for: Diphlorethohydroxycarmalol Derived from Ishige okamurae Improves Behavioral and Physiological Responses of Muscle Atrophy Induced by Dexamethasone in an In-Vivo Model
Source: Pharmaceutics. 2022 Mar 27;14(4):719. doi: 10.3390/pharmaceutics14040719 (PMC9026865; doi:10.3390/pharmaceutics14040719)
Supplement: Supplementary file 1 [file pharmaceutics-14-00719-s001.zip › pharmaceutics-1621825-supplementary.pdf]

pharmaceutics

MDPI

# Supplementary Materials: Diphlorethohydroxycarmalol Derived from *Ishige okamurae* Improves Behavioral and Physiological Responses of Muscle Atrophy Induced by Dexamethasone in an In-Vivo Model

**Bomi Ryu, Seyeon Oh, Hye-Won Yang, Batsukh Sosorburam, Dong-Min Chung, Minyoung Seo, Shin Jae Park, Kyunghee Byun and You-Jin Jeon**

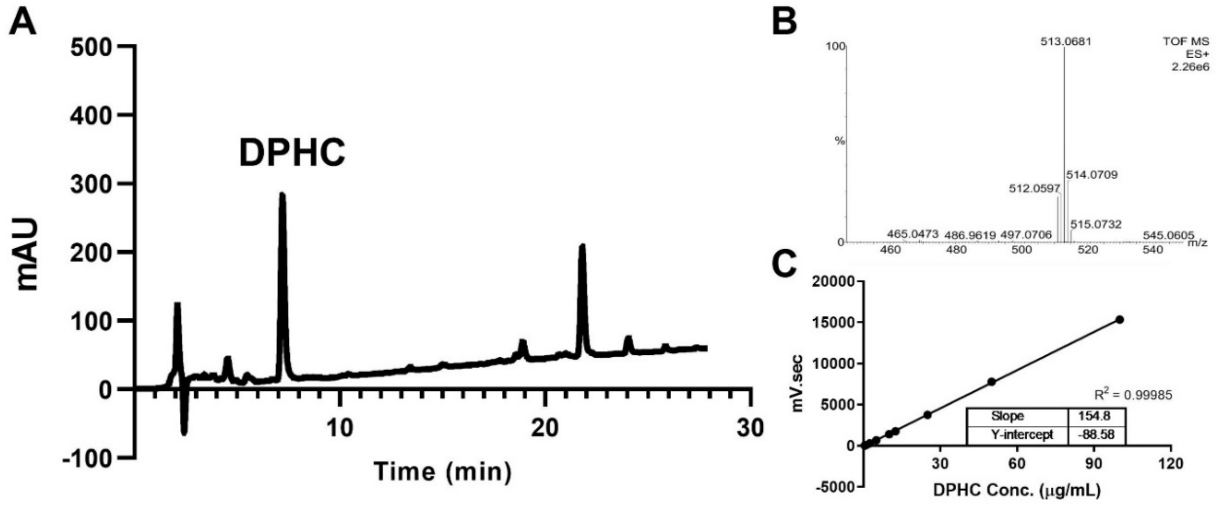


**Figure S1.** HPLC (**A**) and MS/MS (**B**) chromogram data of DPHC in IO and standard curve data for quantification of DPHC (**C**).

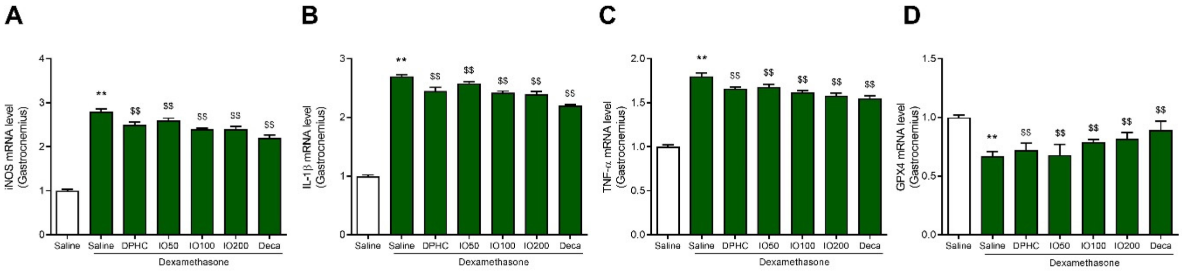


**Figure S2.** Levels of mRNA related to oxidative stress in gastrocnemius muscle of DEX-treated mice displaying muscle atrophy. The panels display results for iNOS (**A**), iL-1β (**B**), TNF-$\alpha$ (**C**), and GPX4 (**D**). Each group was examined in $n = 3$ mice. All values are expressed as the mean ± standard error. Significant differences are indicated as ** $p < 0.01$ compared with control or $^{\$\$}$ $p < 0.01$ compared with DEX-saline (Mann–Whitney U test).

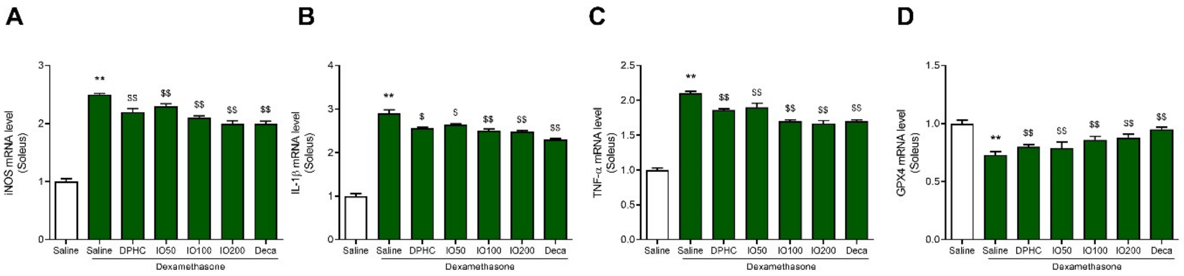


**Figure S3.** Levels of mRNA related to oxidative stress in soleus muscle of muscle DEX-treated mice displaying atrophy. The panels display results for iNOS (**A**), iL-1β (**B**), TNF-$\alpha$ (**C**), and GPX4 (**D**). Each group was examined in $n = 3$ mice. All values are expressed as the mean ± standard error. Significant differences are indicated as ** $p < 0.01$ compared with control or $^{\$}$ $p < 0.05$, $^{\$\$}$ $p < 0.01$ compared with DEX-saline (Mann–Whitney U test).

**Table S1.** List of primer for qRT-PCR.

| **Gene** | | **Primers** |
|---|---|---|
| *Foxo3a* | Forward | 5′-ACC TTC GTC TCT GAA CTC CTT G-3′ |
| | Reverse | 5′-AGT GTG ACA CGG AAG AGA AGG T-3′ |
| *Atrogin-1* | Forward | 5'-GAC ATT CAG AAC AGC AAA ACC A-3′ |
| | Reverse | 5'-GCT CCT TCG TAC TTC CTT TGT G-3′ |
| *MuRF1* | Forward | 5'-ATC TAG CCT GAT TCC TGA TGG A-3′ |
| | Reverse | 5'-ACC ACA GGC TTG GTA AAC ATC T-3′ |
| *PI3k* | Forward | 5′-AGC TGA GTA CCG AGA GAT CGA C-3′ |
| | Reverse | 5′-TCA GCC ACA TCA AGT ATT GGT C-3′ |
| *Akt* | Forward | 5′-CCT TTA TTG GCT ACA AGG AAC G-3′ |
| | Reverse | 5′-GTG CCA CTG AGA AGT TGT TGA G-3′ |
| *TRPV4* | Forward | 5′-CCCATCCTCAAAGTCTTCAATC -3′ |
| | Reverse | 5′- GGTCAACAAGAAGGAGAGCAGT-3′ |
| *A1R* | Forward | 5′-GAAGATTTGGAACGACCACTTC -3′ |
| | Reverse | 5′-AGTCATCAGCTTTCTCCTCTGG -3′ |
| *Myostatin* | Forward | 5′- TTTACCTGTTCATGCTGATTGC-3′ |
| | Reverse | 5′- CATTACACAGCCCCTCTTTTTC-3′ |
| *SIRT1* | Forward | 5′-TGTGTGGAAGAAAAACCACAAG -3′ |
| | Reverse | 5′- ACCAACAGCCTTAAAATCTGGA -3′ |
| *TGF-β* | Forward | 5'-TGA CGT CAC TGG AGT TGT AC-3′ |
| | Reverse | 5′ GGT TCA TGT CAT GGA TGG TG-3′ |
